# Assessment of pressure-volume relations in univentricular hearts: Comparison of obtainment by real-time 3D echocardiography and mini pressure-wire with conductance technology

**DOI:** 10.1371/journal.pone.0246031

**Published:** 2021-02-01

**Authors:** Katharina Linden, Christian Winkler, Johannes Breuer, Ulrike Herberg

**Affiliations:** Department of Pediatric Cardiology, Children’s Hospital, University Hospital Bonn, Bonn, Germany; Scuola Superiore Sant'Anna, ITALY

## Abstract

**Objectives:**

The gold standard to obtain pressure-volume relations (PVR) of the heart, the conductance technology (PVR_Cond_), is rarely used in children. PVR can also be obtained by 3D-echocardiography volume data combined with simultaneously measured pressure data by a mini pressure-wire (PVR_3DE_). We sought to investigate the feasibility of both methods in patients with univentricular hearts and to compare them, including hemodynamic changes.

**Methods:**

We studied 19 patients (age 2–29 years). PVR_3DE_ and PVR_Cond_ were assessed under baseline conditions and stimulation with dobutamine.

**Results:**

Obtaining PVR_3DE_ was successful in all patients. Obtaining PVR_Cond_ was possible in 15 patients during baseline (79%) and in 12 patients under dobutamine (63%). Both methods showed that end-systolic elastance (Ees) and arterial elastance (Ea) increased under dobutamine and that Tau showed a statistically significant decrease. Intraclass correlation (95% confidence interval) showed moderate to good agreement between methods: Ees: 0.873 (0.711–0.945), Ea: 0.709 (0.336–0.873), Tau: 0.867 (0.697–0.942). Bland-Altman analyses showed an acceptable bias with wider limits of agreement: Ees: 1.63 mmHg/ml (-3.83–7.08 mmHg/ml), Ea: 0.53 mmHg/ml (-5.23–6.28 mmHg/ml), Tau: -0,76 ms (-10.73–9.21 ms).

**Conclusion:**

Changes of PVR-specific parameters under dobutamine stimulation were reflected in the same way by both methods. However, the absolute values for these parameters could vary between methods and, therefore, methods are not interchangeable. Obtaining PVR_3DE_ in a single ventricle was easier, faster and more successful than PVR_Cond_. PVR_3DE_ provides a promising and needed alternative to the conductance technology for the assessment of cardiac function in univentricular hearts.

## Introduction

Congenital heart defects (CHD) occur with a prevalence of 107: 10,000 live births of which 29.4% are severe defects requiring early intervention [1]. Univentricular hearts or CHD which are functionally univentricular occur in 2.8% of the 107 [1].

Especially in those patients, the neonatal period until early infancy is the crucial time period for important and far-reaching decisions. Cardiac size and function are especially important factors in the decision making process that will also determine choice of treatment and the resulting clinical outcome. Conventional two-dimensional echocardiography plays an important role but is limited in the sufficient evaluation of systolic and especially diastolic function [2]. Pressure-volume relations (PVR) of the heart do allow assessment of systolic and diastolic function and provide important insight into cardiac physiology and pathophysiology of CHD [3–6].

The considered gold standard to acquire PVR is the conductance catheter technology (Cond). It has been used in basic and clinical research in the left and right heart as well as in univentricular hearts [3, 5, 7–9]. However, the method is rarely used in children due to the invasive procedure of placing a stiff catheter in the ventricle and the need for repeated fluoroscopy to insure correct placement along the axis of the ventricle. Moreover, it is restricted to older age due to the required catheter size. In most cases, the smallest commercially available conductance catheter for human use (4F, dependent on manufacturer requiring a 5F long sheath) is too large for neonates and small infants. Therefore, a less invasive method is needed for infants and small children.

PVR can also be obtained by 3D-echocardiography (3DE) volume data in combination with intraventricular pressure data simultaneously measured by a mini pressure-wire (PVR_3DE_).

The aim of this study was to investigate the feasibility of obtaining PVR_3DE_ and PVR_Cond_ in young patients with univentricular hearts and to compare both methods, including hemodynamic changes.

## Methods

### Patients

19 Patients with univentricular hearts, both single right and left ventricle were included in this study. Patients were in the stages of Glenn- or Fontan-circulation.

In all patients obtainment of PVR_3DE_ and PVR_Cond_ was done during a routine and clinically indicated cardiac catheterization. All catheterizations were performed under general anesthesia. The study was approved by the local ethics institutional review committee "Ethikkommission Universitätsklinikum Bonn" (Registration No. 007/15) and conformed to the principles of the Declaration of Helsinki as well as German law. Written consent was given by the legal guardian or in person by the adult subjects.

### Obtainment of pressure-volume-relations

Obtainment of PVR by 3DE and pressure data from a mini pressure-wire has been used previously by our group. We showed that calculation of PVR_3DE_ is feasible and reproducible [10]. Moreover, we compared PVR_3DE_ and PRV obtained by conductance technology (PVR_Cond_) in small healthy left ventricles of piglets (3.6–8 kg). We found that the combination of 3DE and mini pressure-wire measurements provided a reliable assessment of PVR compared to Cond. Hemodynamic changes were well detected by both methods [11].

For PVR_3DE_ ventricular volume data was obtained by 3D datasets using a matrix transducer (Philips ie33, matrix-transducer X5 and CX7, Andover, USA). Depending on the patient´s weight and clinical situation, a transthoracic (5 patients), transesophageal (11 patients) or substernal approach (3 patients) was used. For data acquisition and data analysis, a standardized protocol was used [10, 12].

**Fig 1:**
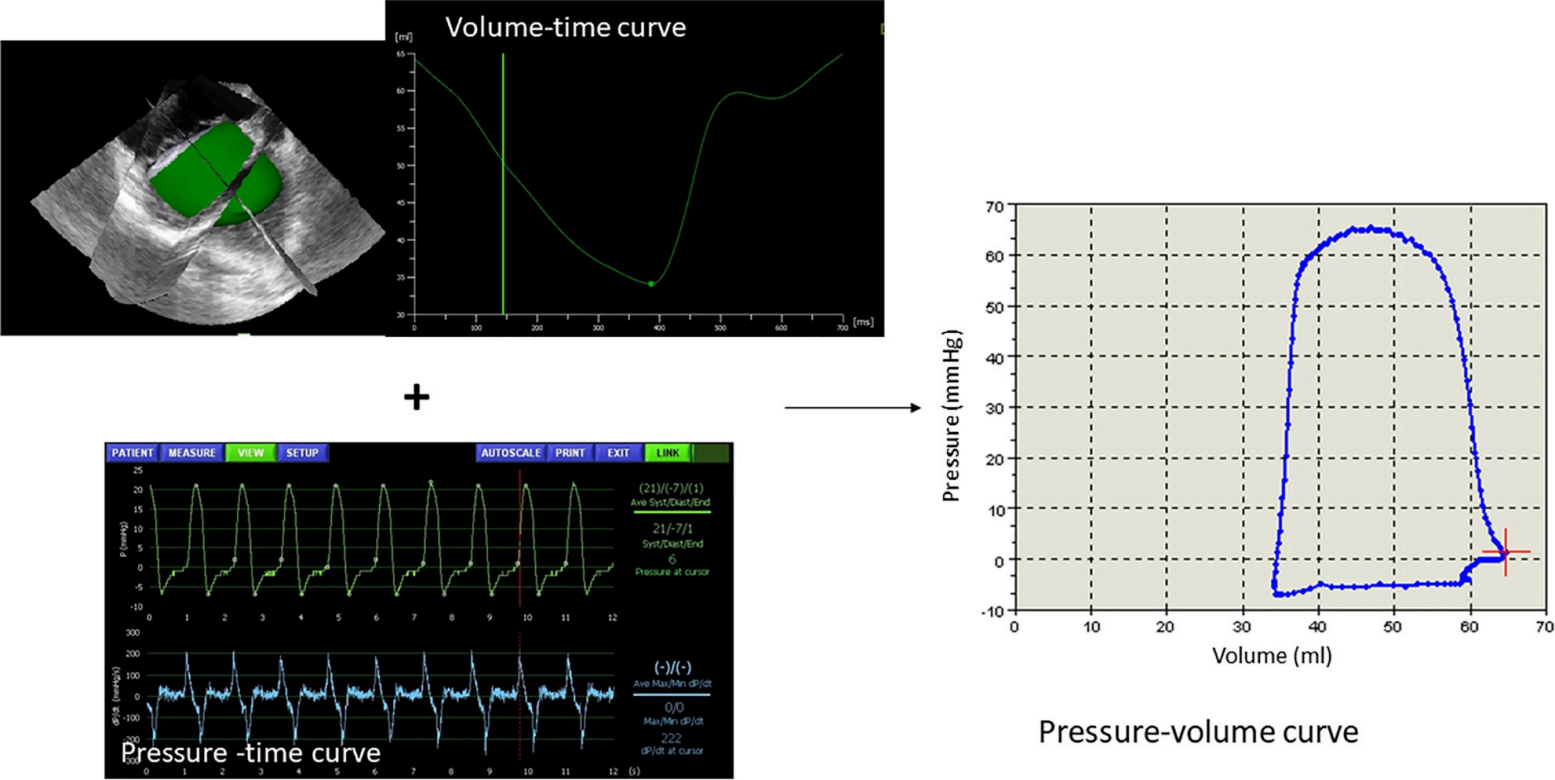
Example of a volume-time curve assessed by three-dimensional echocardiography and the ventricle cast reconstruction. In combination with the simultaneously acquired pressure-time curve a pressure-volume curve is obtained.

3D datasets of the ventricle were acquired during expiratory breath holding (full volume acquisition over 4–7 cardiac cycles). Depending on the depth of penetration, the mean 3D-volume frame rate ± SD was 27.2 ± 4.1 [range: 37.7–19.7] 3D volumes/s; corresponding to 16.7 ± 5.1 [30 – 10] 3D volumes per cardiac cycle respectively. End-diastolic volume (EDV), end-systolic volume (ESV), stroke volume (SV), and ejection fraction (EF) were calculated using TomTec 4D LV-Analysis software 3.1 (Image-Arena version 4.6; Build 4.6.3.9, TomTec, Unterschleißheim, Germany). The apex and the Av-valve were marked. First, the automated border detection traced the contours of the ventricle. Second, manual adjustments to these traced contours were made as needed. A volume-time-curve was then computed by the software (Fig 1).

Continuous monitoring of cardiovascular pressures was performed using a 0.014 French (0.036 mm) mini pressure-wire (PressureWire, Radi, St. Jude Medical, St. Paul, USA). This high-fidelity guidance wire carries a micromanometer at its tip and is attached to an external working station (Radi Analyzer, Software Physiomon 2.0), allowing instantaneous online recording of pressure–time curves. Pressure data were measured with the Radi Analyzer (Software Physiomon, St. Jude Medical, St. Paul, USA) with a sampling rate of 200 Hz. This mini pressure-wire was calibrated and advanced into the ventricle. In general, a microcatheter is suitable for this. Because our study was performed during a routine and clinically indicated cardiac catheterization we used the 3F or 4F sheath and a 3F or 4F pigtail or multi-purpose catheter that was needed for the examination, anyway. In patients with Fontan-circulation an arterial access was necessary. In patients with Glenn-circulation we used a venous access, if possible. If correct insertion of the catheter was not possible an arterial access was used. Continuous monitoring of pressure–time curves allowed assessment of the position of the mini pressure-wire inside the guiding catheter without additional radiation until the pressure-wire was placed properly in the ventricle. To confirm the proper position of the wire within the ventricle in this study, short radiation was used.

For obtainment of PVR_3DE_, full volume 3D datasets and pressure measurements were acquired simultaneously.

Volume time curves, which usually have sampling rates between 330 and 220 Hertz, were synchronized to the pressure-time curve by up sampling through linear interpolation using a customized software. PVR_3DE_ were obtained by the same customized software by combination of the synchronized volume-time curves derived by 3DE and the simultaneously acquired pressure-time curves (Fig 1) [10].

For obtainment of PVR_Cond_ we used a 4F combined pressure/volume conductance catheter with 12 electrodes, spacing 6 mm (CD Leycom, Hengelo, The Netherlands). For insertion of this catheter a 5F sheath is necessary. Before insertion, the catheter was placed into 0.9% saline solution and the pressure sensor was calibrated. Insertion of the catheter into the ventricle was done using fluoroscopic guidance. Signals were displayed by the CFL-M Inca (CD Leycom, Hengelo, The Netherlands). Volume calibration was done in baseline measurements using end diastolic volume (EDV) and end systolic volume (ESV) obtained by 3DE as references [7, 13].

From the PVR_3DE_ and PVR_Cond_, systolic function was measured using end-systolic elastance (Ees), which was calculated by single-beat estimation method [14]. The effective arterial elastance (Ea) was calculated from the ratio of end-systolic pressure (ESP) to stroke volume (SV) [15]. For the assessment of diastolic function we used a single-beat approach calculating the EDV at a common end-diastolic pressure (EDP) of 10.0 mmHg (EDV10) by the formula developed by Klotz in 2006 [16].

The early active relaxation process (decay of the ventricular pressure during isovolumetric relaxation) is reflected by the isovolumetric relaxation constant Tau. Tau was calculated using a logistic fit [17, 18]. Maximal and minimal rate of pressure change over time (dp/dt max and dp/dt min) were determined from continuous pressure measurement. Heart rate (HR), SV, and cardiac index (CI), ESP and EDP were analyzed.

### Study protocol

First, measurements of PVR_3DE_ were done under baseline conditions (BL). Then the mini pressure-wire was removed and the conductance catheter was inserted into the ventricle for baseline measurements of PVR_Cond_. If possible, catheterization of the ventricle was done via a venous access in patiens with a Glenn-circulation. Otherwise an arterial access via the aorta was used. The conductance catheter was left in place and 10 μg/kg/min of dobutamine were administered for 10 minutes until a steady state of heart rate and blood pressure was reached (Dobu). Then measurement of PVR_Cond_ under dobutamine stimulation was performed. The conductance catheter was removed and the mini pressure-wire was inserted. PVR_3DE_ under dobutamine stimulation was assessed. This was done because we observed in previous studies echocardiographic artifacts produced by the conductance catheter, which impaired the semiautomatic contour analysis during 3DE volume calculation. Also, the mini pressure-wire disturbed conductance measurements [11].

### Statistics

Statistical analyses were performed using Graph Pad Prism 5 (GraphPad Software, San Diego, CA, USA) and IBM PSS Version 25 (SPSS Inc, Chicago, USA).

The differences between BL and stimulation with dobutamin were analyzed using paired Student t-tests or Wilcoxon signed-rank tests, as appropriate. Data for Tau, Ees, Ea and EDV10 were log transformed before analysis. Differences were considered to be significant when the p-value <0.05.

Comparison between Tau, Ees, Ea and EDV10 derived from PVR3DE and PVRCond were performed using Bland–Altman analysis and Intraclass correlation (ICC) with one-way random effects.

Data are presented as mean ± standard deviation or median and range.

## Results

We included 19 patients aged 2–29 years in the study. Twelve patients had a single right ventricle and 7 patients a left ventricle.

We were able to acquire PVR_3DE_ in all patients under baseline as well as dobutamine condition. We decided to use the same software for all measurements and choose the software designed for a left ventricle because the single ventricles in our study are systemic ventricles. Moreover, we found that this software allows for the most individual adjustment needed for the individual geometry of single ventricles. In one patient 3DE-data sets under dobutamine could not be analyzed due to a technical defect during acquisition resulting in an insufficient frame rate. The procedure to assess PVR_3DE_ took 5–10 minutes for each condition.

Acquisitions of PVR_Cond_ were not possible in all patients. Placement and measurement took 10 to 30 minutes. In four patients (21%) we were not able to place the conductance catheter correctly so that an adequate measurement was not possible. In another three patients (15.7%) an adequate positioning of the catheter was only given under baseline conditions but not under dobutamine stimulation. One patient had to be excluded due to arrhythmia, although measurements were technically possible.

Therefore, we were able to compare PVR_3DE_ and PVR_Cond_ in 14 patients (Tables 1 and 2). In patients with Glenn-circulation a venous access with satisfactory insertion of the conductance catheter was only possible in one patient. In all other patients an arterial access was used. Comparable measurements under baseline as well as dobutamine conditions were possible in 10 patients. In total, 24 measurements were compared.

**Table 1:** Patient characteristics.

Age [years] median and range	7 [2 – 29]
Weight [kg] median and range	18.95 [10.4–55]
Gender	m = 10
Status Glenn	*n* = 3
	RV = 2
	LV = 1
Status Fontan	*n* = 11
	RV = 9
	LV = 2

RV, right ventricle; LV, left ventricle

**Table 2:** Parameters obtained by 3D-echocardiography combined with mini pressure-wire and conductance technology under baseline and dobutamine stimulation.

	3DE		Cond	
	BL	Dobu	BL	Dobu
SV (ml)	22 ± 12.5	18.6 ± 9	26.8 ±15	25.7 ± 19
CI (l/min/m^2^)	2.1 ± 0.8	2.7 ± 1.3 *	2.5 ± 1	3.4 ± 1.7 *
HR (min^-1^)	84.3 ± 21.8	125.5 ± 19.8 *	83.1 ± 20.8	119.2 ± 23.3 *
ESV (ml)	35.8 ± 25.5	30.8 ± 20.8	28.8 ±15.9	26.5 ±17.9
EDV (ml)	52.3 ± 30.4	45.9 ±27.2	52.2 ±30.6	47 ± 32.3
dp/dt max (mmHg/s)	833.4 ±224.1	2461 ± 719.5 *	742.6 ± 217.4	2321 ± 920.3 *
dp/dt min (mmHg/s)	-1055 ± 296.5	-1908 ± 451.3 *	-978.8 ± 227.8	-1736 ± 492.2 *
ESP (mmHg)	67.9 ± 12.6	90.2 ± 30.5 *	64.8 ± 11.2	96.5 ± 27.4 *
EDP (mmHg)	4.5 ± 4	4.6 ± 4.7	5.9 ± 2.3	5.5 ± 2.8
Ees (mmHg/ml)	3.7 ± 2.1	9.3 ± 7 *	2.5 ± 2	6.6 ± 5.3 *
Tau (ms)	25 ± 5.7	16.1 ± 5.1 *	24.8 ± 6.9	18.5 ±8.2 *
Ea (mmHg/ml)	3.8 ± 2	5.7 ± 2.4 *	3.1 ± 1.7	5.6 ± 4.8 *
EDV10 (ml)	68.5 ± 33.8	55.3 ± 31.2	59 ± 31.6	54.8 ± 32.4

Data are presented as means ± SD. SV, stroke volume; CI, cardiac index; HR, heart rate; ESV, end-systolic volume; EDV, end-diastolic volume; ESP, end-systolic pressure; EDP, end-diastolic pressure; Ees, end-systolic elastance; Ea, arterial elastance; EDV10, EDV at an EDP of 10.0 mmHg; 3DE, 3D-echocardiography combined with mini pressure-wire; Cond, conductance technology

Baseline vs. stimulation with dobutamine:

* P < 0.05

**Fig 2:**
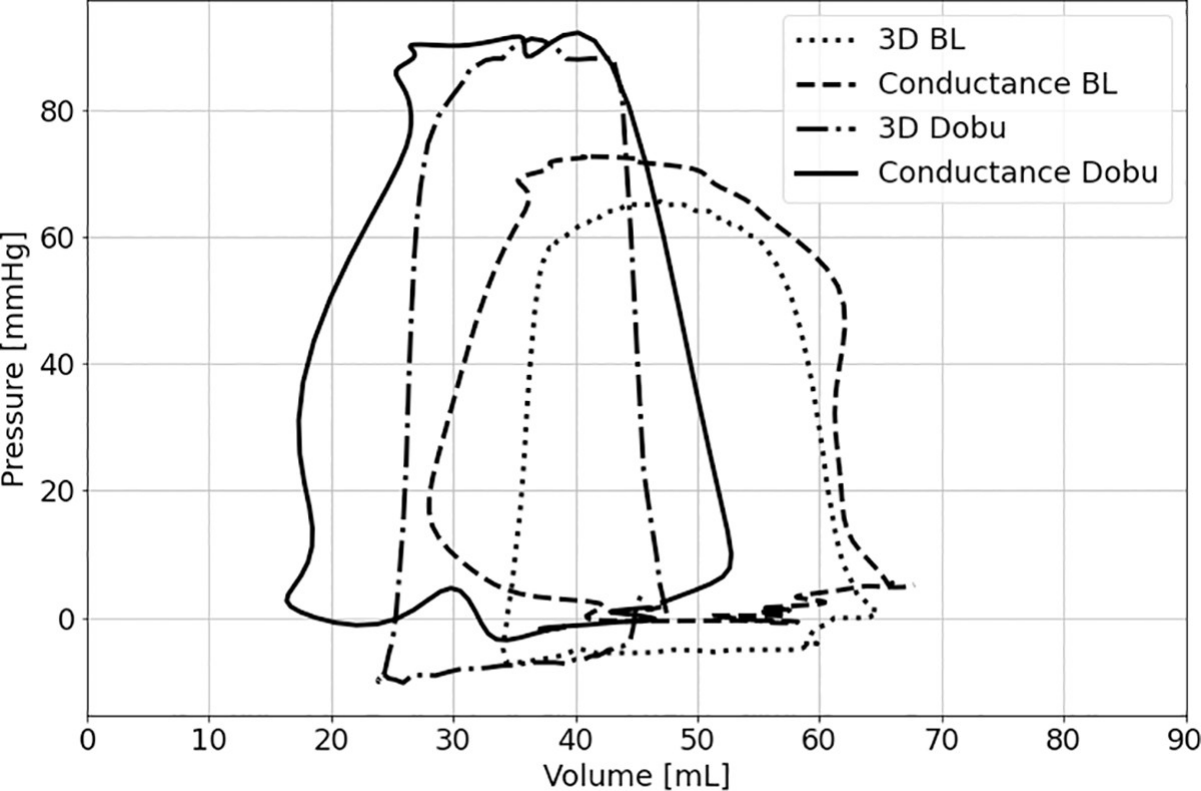
Example of pressure-volume loops of a single right ventricle assessed by 3D echocardiography (3D) and conductance under basal conditions (BL) and stimulation with dobutamine (Dobu).

Fig 2 shows an example of pressure-volume loops of a single right ventricle assessed by 3DE and Cond under BL conditions and stimulation with dobutamine.

We found that Ees showed a statistically significant increase under stimulation with dobutamine in both measurement techniques. Ea showed an increase under stimulation with dobutamine in both measurement techniques as well. Furthermore, we saw an increasing CI mainly due to an increased heart rate while SV did not differ significantly under dobutamine stimulation (Table 2).

**Fig 3:**
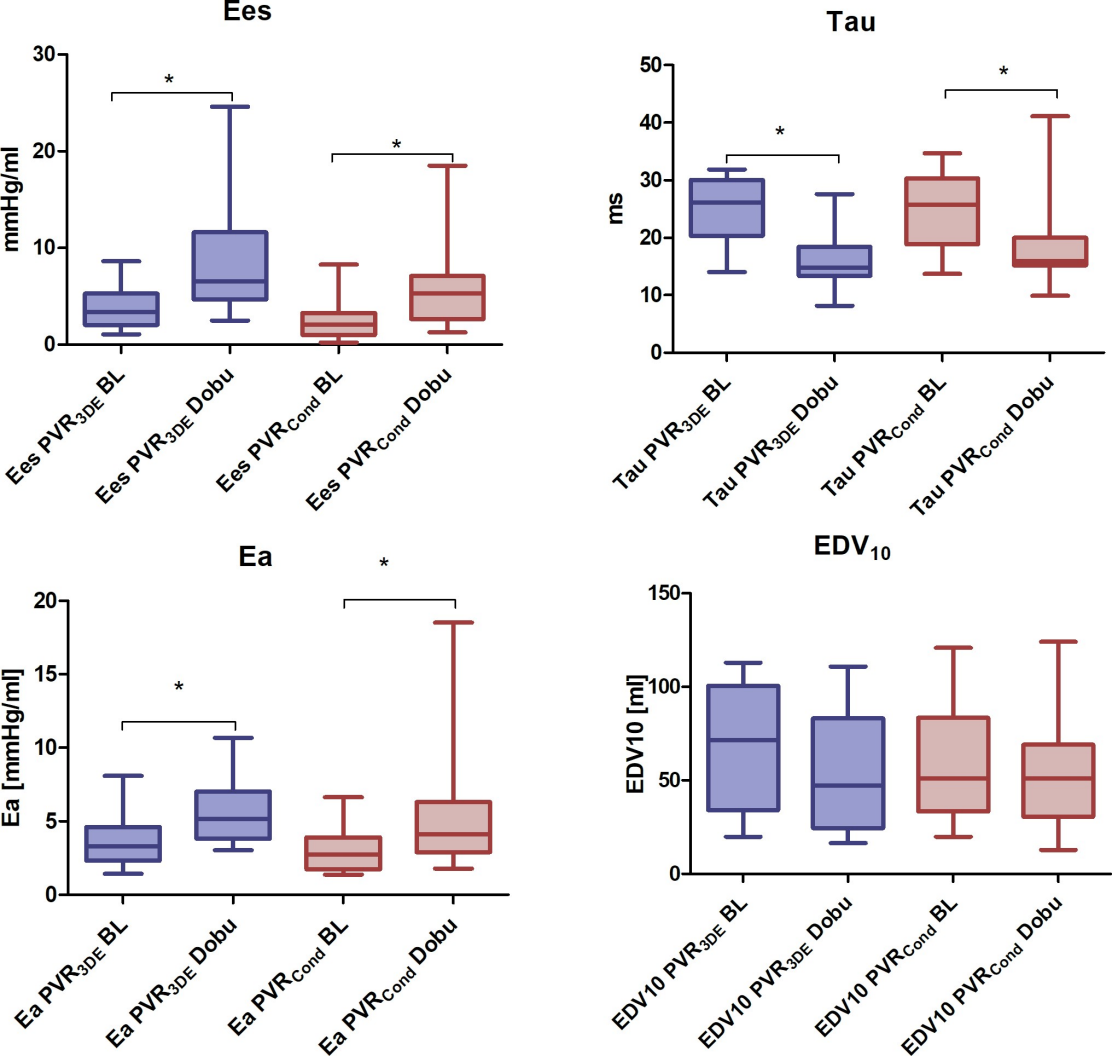
Boxplot of PVR-specific parameters under baseline conditions (BL) and stimulation with dobutamine (Dobu). PVR_3DE_ = 3D-echocardiography combined with mini pressure-wire; PVR_Cond_ = conductance technology; Ees = end-systolic elastance, Ea = effective arterial elastance, EDV10 = EDV at an EDP of 10.0 mmHg Baseline (BL) vs. stimulation with dobutamine (Dobu): * P < 0.05.

The maximal rate of diastolic pressure decay, dp/dtmin, increased under dobutamine. Tau, showed a statistically significant decrease under stimulation with dobutamine. The mean EDV10 decreased under stimulation with dobutamine but this decrease was statistically not significant in either measurement technique. (Fig 3).

Comparing the measurements by 3DE and Cond by Bland-Altman analysis showed a mean bias and limits of agreement of 1.63 mmHg/ml (-3.83–7.08 mmHg/ml) for Ees, -0.76 ms (-10.73–9.21 ms) for Tau, 0.53 mmHg/ml (-5.23–6.28 mmHg/ml) for Ea and 5.74 ml (-27.25–38.73 ml) for EDV10 (S1 Fig).

ICC and their 95% confidence interval was 0.873 (0.711–0.945) for Ees, 0.867 (0.697–0.942) for Tau, 0.709 (0.336–0.873) for Ea and 0.916 (0.8–0.965) for EDV10.

S2 Fig shows the absolute values for Ees measured by 3DE and Cond under BL and Dobu.

## Discussion

In this study, we assessed PVR in univentricular hearts with two different methods: Volume data from 3DE combined with pressure data from a mini pressure-wire and the conductance technology. We compared these two methods in regard to feasibility and parameters obtained from PVR under hemodynamic changes.

We could show that obtaining PVR_3DE_ was possible in all patients while the measurement with Cond was less successful, mostly due to the difficulty of placing the relatively stiff catheter in a correct position in anatomically atypically formed ventricles. In some patients, the adequately positioned conductance catheter under baseline conditions came into an inadequate position under administration of dobutamine. This observation of about 21% unsuccessful placement and/or insufficient signal quality is in accordance with previously reported data of a conductance catheter study in univentricular hearts [3]. It shows that the use of the conductance technique in univentricular hearts is not always easy to perform. In comparison to the obtainment of PVR_3DE_, PVR_Cond_ was associated with a longer examination time and more radiation that was needed for the placement of the catheter.

In previous studies we could show that reproducibility of PVR_3DE_ was good with an interobserver coefficient of variation below 10% [10, 11].

We found that Ees as a parameter for systolic function and contractility and Ea as a measure of systemic afterload showed a statistically significant increase under stimulation with dobutamine. Our values for Ees and Ea measured by Cond are comparable to those of Schlangen et al. in their conductance catheter study in univentricular hearts [3, 19]. We also observed the same response to stimulation with dobutamine with increased CI and HR and constant or even reduced SV as did Schlangen et al. [3]. Other studies also showed that dobutamine affected the heart rate more than the stroke volume, leading to the assumption that there is a limited ability to augment stroke volume in patients after Fontan palliation [20, 21]. The relaxation constant Tau, reflecting the early relaxation process, decreased as an expected expression of a positive lusitropic effect of dobutamine. As another effect of dobutamine we saw that the maximal rate of diastolic pressure decay, dp/dtmin, which indicates the relaxation ability of the ventricle, increased. We also saw a trend towards lower values for EDV10 as a measure of diastolic ventricular function and stiffness. These findings are in accordance with the study of Schlangen et al. and their results for change in Tau and end-diastolic stiffness (eed) [3].

### Comparison 3DE and Cond

Comparing Ees, Tau, Ea and EDV10 we found that both methods reflected changes between baseline and stimulation with dobutamine in the same way. When comparing the absolute values for these parameters, ICC and its 95^th^ confidence interval showed good to moderate agreement [22]. The Bland-Altman analysis revealed acceptable bias, with rather wide limits of agreement. Absolute values varied partially between the methods. This is also reflected in S2 Fig. Therefore, the two methods cannot be regarded as interchangeable [23]. For the longitudinal follow up of one patient, the same method should be used.

In our previous study in which we compared PVR_3DE_ and PVR_Cond_ in small healthy biventricular porcine hearts we found a better agreement between methods [11]. One reason might be the atypically formed single ventricles. The conductance technique assumes a homogenous electrical field throughout the heart chamber and also assumes an elliptically shaped ventricle. Some studies showed that volume measurements by Cond are adequate in a normal right ventricle, even though there is a significant difference in geometry and trabeculation compared to a left ventricle [24, 25]. The shape of univentricular hearts, both left and right ones, can highly differ from regular ventricles. While it is possible to capture these shapes by 3DE, there might occur inaccuracies in the measurements of volume change by Cond.

### Limitations

For technical reasons as explained above, PVR_3DE_ and PVR_Cond_ could not be obtained simultaneously. Therefore, slight hemodynamic changes e.g. non-identical pressures between 3DE and Cond measurements are possible.

To achieve satisfactory temporal and spatial resolution especially in infant hearts, 3D full- volume datasets are needed. They are recorded over 4 cardiac cycles. In consequence, beat-to-beat variability cannot be assessed. The existing technique of single-beat acquisition of 3D datasets does not provide adequate temporal and spatial resolution for the calculation of beat-to-beat variability in the pediatric heart [26]. The current development of new echo transducers with high temporal and special resolution will simplify single beat data acquisition in future.

We calibrated the conductance derived baseline volumes with volumes derived by 3DE. Our ethical committee did not allow for additional measurements by thermodiluation or cardiac magnetic resonance. We cannot rule out that this might have led to bias. Basing the calibration of baseline volumetric data on the same reference in the beginning enabled us to study the tracking of hemodynamic changes of the two methods without an additional bias of a third volumetric method.

For assessment of load-independent variables, we desired a preload-reduction by an inflated balloon-catheter in the vena cava inferior or Fontan-tunnel. Unfortunately, our ethical committee rejected the request for this additional procedure.

Another limitation is the relatively small sample size of this study due to the invasive procedure

### Clinical outlook

Assessment of systolic and diastolic ventricular function in patients with univentricular hearts is needed and can help with decisions on pharmacological treatment. 3DE is available in most centers for pediatric cardiology and the placement of the pressure-wire without radiation can even take place outside of a catheter laboratory. In general, the pressure-wire can be left in place for a while (under heparinization) to perform testing like volume load or the effect of drugs like inotropic agents or vasodilators.

## Conclusion

We found that hemodynamic changes between baseline and stimulation with dobutamine were reflected in the same way by PVR-specific parameters measured by both, PVR_3DE_ and PVR_Cond_. However, the absolute values for these parameters could vary between methods and, therefore, methods are not interchangeable. Obtaining PVR_3DE_ in a single ventricle was more feasible, faster and less invasive regarding size of sheath and radiation than obtaining PVR_Cond_. Consequently, PVR obtained by 3DE and mini pressure-wire provide a promising and needed alternative to the conductance technology for the assessment of cardiac function in univentricular hearts.

## Supporting information

S1 Fig Bland Altman-Plots comparing the measurements by 3D echocardiography and conductance technique. 3DE = 3D echocardiography, Cond = conductance technology, Ees = end-systolic elastance, Ea = effective arterial elastance, EDV10 = EDV at an EDP of 10.0 mmHg, dotted lines = bias and limits of agreement.
(TIF)

S2 Fig Example of the absolute values of end-systolic elastance (Ees) measured by 3D-echocardiography combined with mini pressure-wire (3DE) and conductance technology (Cond) under baseline and dobutamine stimulation. Points = baseline 3DE; circles = Dobu 3DE; filled squares = baseline Cond; open squares = Dobu Cond.
(TIF)
