# Supplementary figures and images for: Assessment of pressure-volume relations in univentricular hearts: Comparison of obtainment by real-time 3D echocardiography and mini pressure-wire with conductance technology
Source: PLoS One. 2021 Feb 1;16(2):e0246031. doi: 10.1371/journal.pone.0246031 (PMC7850469; doi:10.1371/journal.pone.0246031)

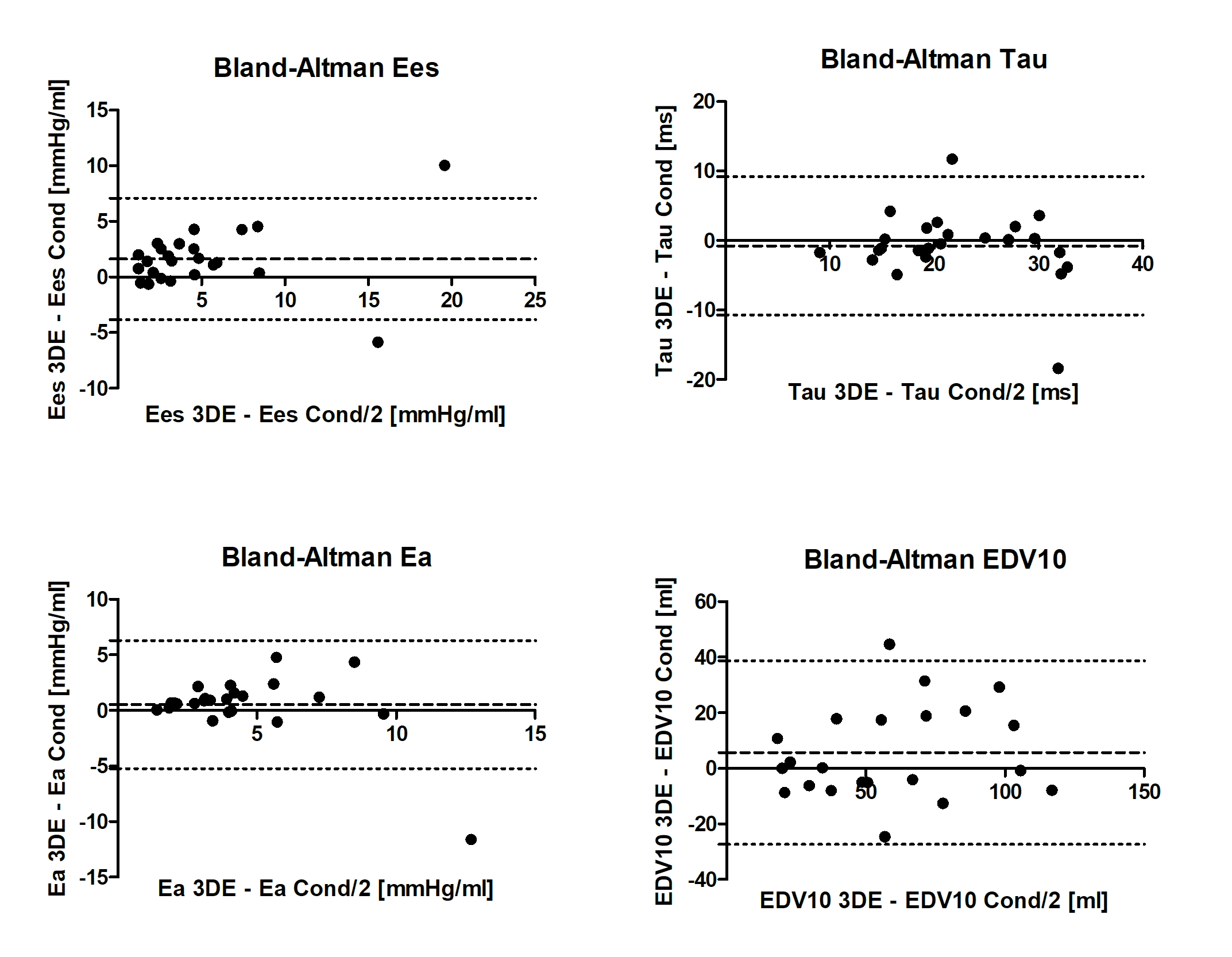

Supplement: S1 Fig — 3DE = 3D echocardiography, Cond = conductance technology, Ees = end-systolic elastance, Ea = effective arterial elastance, EDV10 = EDV at an EDP of 10.0 mmHg, dotted lines = bias and limits of agreement. (TIF) [file pone.0246031.s001.tif]

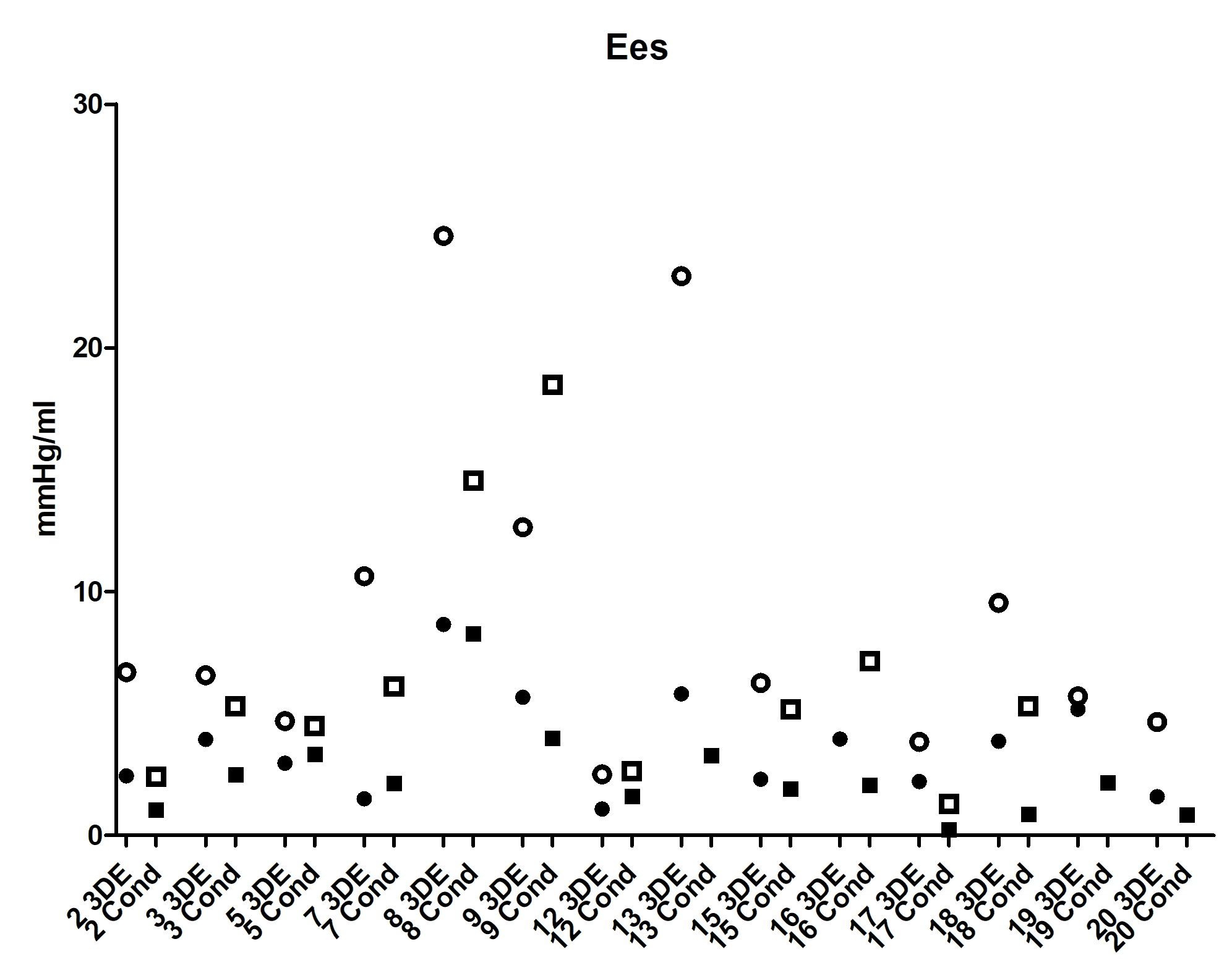

Supplement: S2 Fig — Points = baseline 3DE; circles = Dobu 3DE; filled squares = baseline Cond; open squares = Dobu Cond. (TIF) [file pone.0246031.s002.tif]
